# Assessment of Ready-to-Use Freeze-dried Immobilized Biocatalysts as Innovative Starter Cultures in Sourdough Bread Making

**DOI:** 10.3390/foods8010040

**Published:** 2019-01-21

**Authors:** Ioanna Mantzourani, Antonia Terpou, Athanasios Alexopoulos, Eugenia Bezirtzoglou, Stavros Plessas

**Affiliations:** 1 Laboratory of Microbiology, Biotechnology and Hygiene, Faculty of Agriculture Development, Democritus University of Thrace, Orestiada 68200, Greece; ant.terpou@gmail.com (A.T.); alexopo@agro.duth.gr (A.A.); empezirt@agro.duth.gr (E.B.); 2 Department of Chemistry, University of Patras, Patras 26500, Greece

**Keywords:** *Lactobacillus paracasei* K5, wheat grain sourdough, bread, spoilage, GC/MS

## Abstract

In the present study the effect of innovative biocatalysts as starter cultures in sourdough bread making was explored. The biocatalysts consisted of *Lactobacillus paracasei* K5 and *Lactobacillus bulgaricus* ATCC 11842 (in single and mixed form), immobilized on delignified wheat bran (DWB), and freeze dried without cryoprotectants. The parameters monitored were physicochemical characteristics, mold and rope spoilage appearance, volatile composition, and organoleptic characteristics. Results obtained showed that both biocatalysts exhibit good fermentative activity. However, the best results were achieved when freeze-dried immobilized *L. paracasei* K5 was applied as a single culture. In particular, the produced bread had a higher acidity (8.67 mL 0.1 N NaOH) and higher organic load (2.90 g/kg lactic acid and 1.11 g/kg acetic acid). This outcome was the main reason why this bread was preserved more regarding mold spoilage (14 days) and rope spoilage (12 days), respectively. In addition, the employment of freeze-dried immobilized *L. paracasei* K5 led to bread with better aromatic profile in terms of concentrations and number of volatile compounds produced as gas chromatography/mass spectrometry (GC/MS) analysis proved. Finally, no significant differences were observed through sensorial tests. Last but not least, it should be highlighted that the used microorganisms were cultured in cheese whey, minimizing the cost of the proposed biotechnological procedure.

## 1. Introduction

Nowadays, consumers are very interested in selecting novel or traditional foods containing less or no chemical preservatives [1,2,3]. Likewise, this new trend has been recently developed in the bread industry, particularly through sourdough applications [4]. The use of sourdough in bread making has its roots in antiquity, while currently an upsurge of interest in sourdough applications has been revived [5,6]. The use of sourdough covers these consumers’ demands since it is free of preservatives and offers significant advantages such as higher preservation times, enhanced aromatic profile, increased nutritional value, and health benefits [7,8,9,10,11]. In general, the main reason for all the aforementioned is that through sourdough fermentation, various enzymes, such as amylases, proteases, hemicellulases, and phytases are activated and decreased or increased levels of compounds/metabolites lead to positive effects [12]. However, the different fermentation processes along with the variable microbiota of sourdough, make it a complex matrix that occasionally prohibits the functionality of microorganisms and their desirable metabolites to be released [13]. Likewise, control of some fundamental parameters is required for the production of an effective sourdough such as the proper selection of defined microorganisms (starter cultures), water proportion, type of cereal flour, fermentation time, and temperature [14]. Several scientific reports have been published in the literature proving that the principal applied microbial group for sourdough fermentation are lactic acid bacteria (LAB), due to their natural presence in sourdough microbiota [7,15,16]. 

On the other hand, storage of microorganisms for long time periods prior to their use is required by the food industry and consequently microorganism preservation is an industrial prerequisite [17,18,19]. Microencapsulation seems an attractive method for the food industry in order to guarantee long-term delivery of stable cultures in terms of viability and functional activities [20,21]. Microencapsulation of starter cultures can be conducted with various methods such as spray-drying [22], fluid bed coating [23], and freeze- or vacuum-drying [24,25]. The use of freeze-dried starter bacterial cultures or probiotics is of rising popularity as they can be applied directly in large scale products without any preparatory efforts [18,25,26]. 

Based on the above results, the aim of this study was to evaluate the use of two novel freeze-dried immobilized biocatalysts applied for sourdough bread making: *Lactobacillus paracasei* K5 and *Lactobacillus bulgaricus* ATCC 11842. The main reason for this selection was that a commercially available sourdough starter should contain at least one heterofermentative and one homofermentative LAB in order to assure good acidification and aromatization [27]. Likewise, besides the facultative heterofermentative *L. paracasei* K5, the homofermentative *L. bulgaricus* ATCC 11842 [28] was also selected, in order to produce various types of sourdough breads [29]. In addition, both microorganisms have been successfully applied in sourdough bread making previously [7,30]. Therefore, the main target of the present study was to examine *L. paracasei* K5 and *L. bulgaricus* ATCC 11842 immobilized on wheat bran and freeze dried to be applied as ready-to-use synbiotic biocatalysts in single or mixed forms for sourdough bread making. The parameters determined were (i) physicochemical characteristics, (ii) shelf-life of breads, and (iii) aroma-related compounds. In addition, preliminary sensorial tests were employed.

## 2. Materials and Methods

### 2.1. Microorganisms

The homofermentative *L. delbruekii* ssp. *bulgaricus* (DSMZ, strain ATCC11842) and the novel strain *L. paracasei* K5 recently isolated from Greek feta-type cheese were grown in MRS (de Man, Rogosa and Sharpe) broth (Fluka, Buchs, Switzerland) at 37 °C for 24 h [7]. Production of cell biomass was made through addition of harvested biomass in 2 L of cheese whey and incubation at 30 °C for about 24 h [30]. Suitable amounts of harvested cell biomass were then obtained. Baker’s yeast was a commercial *Saccharomyces cerevisiae* strain obtained in the form of pressed blocks (70% *w*/*w* moisture), manufactured by S.I. Lesaffre, France.

### 2.2. Preparation of the Immobilized Synbiotic Biocatalysts

Wheat bran (WB), was supplied by a local industry (Orestiada, Greece) and was used as an immobilization carrier of the strains *L. paracasei* K5 and *L. bulgaricus* ATCC 11842. Wheat bran consisted approximately of 50% dietary fiber, 20% protein, 7% ash and 4% lipids [31]. Initially, WB was sterilized by autoclaving at 120 °C, 1–1.5 atm for 15 min. Immobilizations were accomplished by mixing (i) 5 g of WB with 0.5 g of harvested *L. paracasei* K5 cell mass (dry weight) and (ii) 5 g of WB with 0.5 g of harvested *L. bulagaricus* ATCC 11842 in 500 mL MRS broth and incubating at 37 °C for 48 h. The two immobilized biocatalysts were washed twice with 1/4 strength Ringers solution targeting removal of free cells and freeze dried overnight in a freeze-drying system (FreeZone 4.5, Labconco, Kansas City, MO, USA) [31]. 

For the enumeration of the immobilized cells, 10 g of each freeze-dried immobilized biocatalyst were blended with 90 mL of sterile 1/4 strength Ringers solution. The suspension was serially diluted (ten-fold), plated on MRS agar (Fluka, Switzerland), and incubated at 37 °C for 48 h. Cell counts were expressed as log CFU/g of wheat bran [31]. Each of the immobilized synbiotic biocatalysts contained about 8 log CFU/g. Both freeze-dried immobilized biocatalysts were employed as starter cultures for sourdough bread production. 

### 2.3. Microbiological Analysis of Sourdoughs

20 g of duplicate sourdough samples were homogenised in 200 ml of phosphate buffer (1.25 mL of 0.25 M solution of KH_2_PO_4_/L distilled water) and subjected to serial dilutions. Yeasts were determined on malt agar (Fluka, Switzerland) after incubation at 30 °C for 3 days and lactobacilli on MRS agar (Fluka, Switzerland) after incubation at 37 °C for 3 days [32].

### 2.4. Sourdough Bread Making 

Commercial white flour was used for bread making (Hellenic Biscuit CO SA, Greece), with the following composition (% *w*/*w*): protein 11.0, carbohydrates 72.0, fat 1.5, fiber 2.2, and moisture 12.0. During bread making, mixing of ingredients was performed mechanically and the dough was molded manually in 1.5 L baking pans.

Six mother sponges were prepared. In particular, two sourdoughs were prepared by mixing 300 g wheat flour and 160 ml tap water with 0.5% and 1% *w*/*w* (on flour basis) freeze-dried immobilized *L. paracasei* K5, respectively, for 15 min. In the same manner, two mother sponges were also prepared by mixing 300 g wheat flour and 160 mL tap water with 0.5% and 1% *w*/*w* (on flour basis) freeze-dried immobilized *L. bulgaricus* ATCC 11842, respectively, for 15 min. Finally, two more sponges were prepared by mixing 300 g wheat flour and 160 mL tap water with freeze-dried immobilized *L. bulgaricus* ATCC 11842 and *L. paracasei* K5 in a ratio of 0.25% and 0.25% *w*/*w* (on flour basis), as well as in a ratio of 0.5% and 0.5% *w*/*w* (on flour basis), respectively. All the sponges were incubated at 30 °C for 24 h. 

Sourdoughs were prepared by mixing 250 g of the aforementioned fermented mother sponges with 300 g wheat flour and 160 mL tap water for 15 min, respectively, followed by incubation at 30 °C for 24 h. Likewise, six sourdoughs were prepared containing: (i) 0.5% (Sourdough A) and 1% (Sourdough B) *w*/*w* (on flour basis) freeze-dried immobilized *L. paracasei* K5, (ii) 0.5% (Sourdough C) and 1% (Sourdough D) *w*/*w* (on flour basis) freeze-dried immobilized *L. bulgaricus* ATCC 11842, (iii) 0.25% (Sourdough E) *w*/*w* (on flour basis) freeze-dried immobilized *L. paracasei* K5 with 0.25% *w*/*w* (on flour basis) freeze-dried immobilized *L. bulgaricus* ATCC 11842, and (IV) 0.5% *w*/*w* (on flour basis) freeze-dried immobilized *L. paracasei* K5 with 0.5% *w*/*w* (on flour basis) freeze-dried immobilized *L. bulgaricus* ATCC 11842 (Sourdough F).

Afterwards, six sourdough breads were produced containing 30% *w*/*w* (on flour basis) of the aforementioned sourdoughs. Specifically, the following six breads were produced by: (i) sourdough A (LP1), (ii) sourdough B (LP2), (iii) sourdough C (LB1), (iv) sourdough D (LB2), (v) sourdough E (LPB1), and (vi) sourdough F (LPB2). The doughs of all the breads contained 150 g of each sourdough, 500 g wheat flour, 270 mL tap water, and 4 g salt. In all cases an amount of 1% *w*/*w* (on flour basis) of pressed baker’s yeast was added. All doughs were fermented at 30 °C for 2 h, proofed at 40 °C for 60 min, and baked at 230 °C for approximately 40 min [7].

Control trials were carried out with sourdough prepared with traditional, wild microbiota provided by a local bakery. The bread (W) produced contained 30% (on flour basis) of a traditional wild microbiota sourdough. The recipe and the procedure followed was the same as described above for the aforementioned sourdoughs. All trials were carried out in triplicate.

### 2.5. Organic Acids Analysis

10 g of dough were mixed with 90 mL of sterile distilled water for 2 min using a stomacher blender (Stomacher 400, Seward Laboratory, London, UK). The sourdough bread extracts were centrifuged at 20,000 rpm and organic acids (lactic, acetic, formic, propionic, *n*-valeric, and caproic) were determined by ion-exchange liquid chromatography as described previously by Plessas et al. [30]. Determinations of all organic acid concentrations were carried out by means of standard curves.

### 2.6. Determination of pH and Total Titratable Acidity

The pH and total titratable acidity (TTA) values of sourdough bread samples were determined as described previously by Mantzourani et al. [7]. The TTA was expressed as the volume (mL) of 0.1 N NaOH consumed.

### 2.7. Determination of Specific Loaf Volume

The loaves were weighed, and the loaf volume was measured by the rapeseed displacement method [30]. The loaf volumes were calculated by deducting the rapeseed volume from the container volume. The specific loaf volume was calculated as mL/g.

### 2.8. Analysis of Aroma Volatiles

Gas chromatography/mass spectrometry (GC/MS) analysis of volatile compounds was carried out using the headspace solid-phase microextraction (SPME) sampling technique as described previously by Plessas et al. [33]. Volatile compounds were identified by comparison with standard compounds (Sigma-Aldrich, St. Louis, MO, USA) and MS data with those in NIST107, NIST21, and SZTERP libraries. For semi-quantitative analysis of volatiles, 4-methyl-2-pentanol (Sigma-Aldrich) diluted in pure ethanol was used as the internal standard (IS) at various concentrations (4, 40, and 400 μg/g of sample). The volatile compounds were quantified by dividing the peak areas of the compounds of interest by the peak area of the IS and multiplying this ratio by the initial concentration of the IS (expressed as μg/g). All assays were carried out in triplicate.

### 2.9. Rope and Mold Spoilage

Sourdough bread samples of similar shape and size were cut from the same loaf of bread and stored at room temperature. The appearance of rope spoilage was conducted through daily macroscopic evaluation of the main spoilage characteristics such as distinct flavor of ripe cantaloupe, discoloration, and sticky threads [34]. Regarding mold spoilage evaluation, the surface of each sample was macroscopically observed daily for visible fungi colonies [30].

### 2.10. Preliminary Sensory Evaluation

The sourdough breads were daily assessed at a local bakery for a total of five days through blind sensory evaluation test. Specifically, 20 randomly untrained testers (consumers) evaluated the produced breads giving scores between 0 (unacceptable) and 10 (exceptional) for attributes of flavor, taste, and overall quality such as volume, texture, and color [30]. At least three samples were provided to each tester. The results were recorded as average scores plus standard deviations. 

### 2.11. Statistical Analysis

In order to assess significant differences among sourdough bread samples, the effects of the different sourdough starters on bread physicochemical characteristics, aroma volatile compounds, appearance of mold and rope spoilage, and sensorial analysis were analyzed for their mean differences with the Analysis of Variance (ANOVA) procedure followed by Duncan’s post hoc multiple range test to extract the specific differences between the various treatments. Analysis was performed by using IMB SPSS (version 20, IBM Corp., Armonk, NY, USA) at an alpha level of 5%. 

## 3. Results

Freeze-dried immobilized *L. paracasei* K5 and *L. bulgaricus* ATCC 11842 as single and mixed cultures were employed for sourdoughs preparations and then assessed in sourdough bread making. A traditional sourdough was also applied in sourdough bread making (control). All sourdoughs were added in concentrations of 30% *w*/*w* (flour basis) in bread dough. Initially, all sourdoughs were microbiologically analyzed for LAB and yeasts levels (Table 1). Sourdough breads were analyzed for pH, TTA and specific loaf volume, lactic, acetic acid and other organic acids (Table 2), mold and rope spoilage (Figure 2), and aroma volatile compounds (Table 3). The breads were also subjected to consumer sensory evaluations (Table 4). 

### 3.1. Microbiota of Sourdoughs 

Initially, microbiological analysis was conducted in all sourdoughs prepared regarding levels of LAB and yeasts and the results are shown at Table 1. No statistically significant differences were observed between sourdoughs prepared with the addition of total 0.5% starter culture (Sourdoughs A, C, F) and the addition of total 1% starter culture (Sourdoughs B, E, F). In addition, control sourdough contained approximately the same levels of LAB and yeasts as the other sourdoughs.

### 3.2. Sourdough Bread Quality Characteristics

Specific loaf volume of all sourdough bread samples varied in respectable values (above 2 mL/g), while no statistically significant differences were observed regarding pH values, except that all samples had lower values than the control one (W) (Table 1). However, higher TTA (*p* < 0.05) value was determined in LP2 (8.67 mL 0.1 N NaOH), followed by LB1 and LB2 samples. The same outcome was observed in the case of organic acids analysis (Table 2). Particularly, LP2 contained higher amounts (*p* < 0.05) of lactic and acetic acid, as well as formic, propionic, *n*-valeric, and caproic acid compared to all the other samples analyzed, giving an obvious explanation about its higher levels of acidity.

### 3.3. Volatile Compounds 

Results obtained through the semi-quantitative determination of volatile compounds from the sourdough bread samples (LP2, LB2, LPB2, and W) by GC/MS analysis are presented in Table 3. Key volatile compounds such as heptanol, 2-phenylethyl acetate, hexanal, 1-octen-3-ol, benzaldehyde, 2-nonenal, and furfural were identified [33,35,36]. Regarding the main chemical groups of volatile compounds (alcohols, esters, and carbonyl compounds), statistically significant differences were observed. Particularly, LP2 contained more alcohols (12), esters (13), and carbonyl compounds (11) than all the other sourdough bread samples studied (in total 36 volatile compounds). The second better sample, based on the number of volatile compounds determined was LPB2 (31 volatile compounds), while LB2 contained 27, and W 24, respectively. 

**Figure 1:**
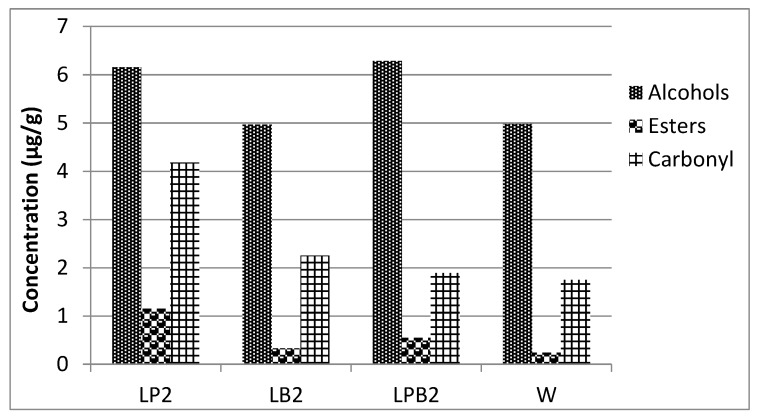
Concentrations (μg/g) of certain volatile chemical groups expressed as mean values in the sourdough bread samples.

It should be underlined that even though the presence of a volatile compound in a food matrix does not convey certain positive influence, increased numbers of volatile compounds can exhibit higher aroma complexity that influences positively the aroma of bread [10]. In addition, LP2 contained higher concentrations of esters and carbonyl compounds (1.15 μg/g and 3.77 μg/g, respectively) compared to all the other bread samples (Figure 1), which is considered a positive outcome since high numbers and concentrations of esters are required for the achievement of good aroma in bread [37]. 

### 3.4. Evaluation of Spoilage

**Figure 2:**
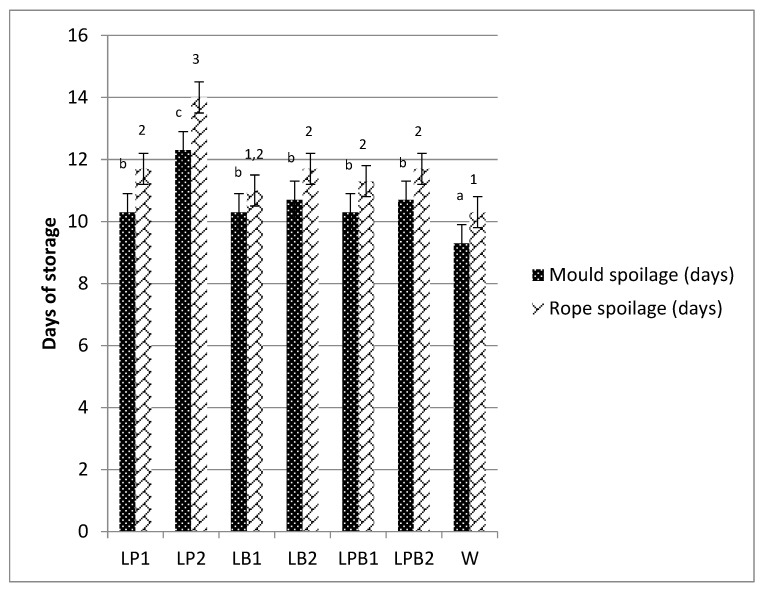
Resistance of sourdough bread samples against rope and mold spoilage expressed as days plus standard deviations. Similar lower-case letters (for mold spoilage) and figures (rope spoilage) indicate homogenous groups (ANOVA with Duncan post hoc application in 95% significance level).

The appearance of mold and rope spoilage in all sourdough bread samples was assessed through daily macroscopic observations (Figure 2). 

There was a statistically significant difference in all sourdough bread samples time of storage for mold (ANOVA F:10.29, *p* < 0.05) and rope spoilage (ANOVA F:16.53, *p* < 0.05) appearance. Regarding mold spoilage appearance, LP2 was more resistant (*p* < 0.05) compared to all other samples since it was spoiled at the 14th day. LP1 was spoiled at the 12th day of storage, while most of the other samples were spoiled at the 10th day, except for W (at the 6th day). The prolonged shelf-life of LP2 can be attributed to the higher concentrations of organic acids, especially lactic and acetic acid. At this point, the effect of acetic acid should be underlined. Particularly, addition of acetic acid to bread delays fungal spoilage and in concentrations of 10–30 mmol/kg has also a beneficial impact on bread flavor [36]. In addition, it has been reported that the levels of acetic acid which accumulated during the fermentation process affected the rate of the development of fungi on the bread, and that the lactic acid does not produce a significant effect [38]. Interestingly, other organic acids may also have antifungal activities. Formic, propionic, together with caproic and acetic acids, were the principal mold inhibitor in sourdough fermentation through application of homo- and heterofermentative LAB [39]. Likewise, the higher organic acid load of LP2 seems to be the explanation for the delay of mold spoilage appearance.

In addition to their antifungal capacity, LAB also produce organic acids and effective antibacterial compounds [40]. Therefore, concerning rope spoilage, LP2 developed spoilage at the 14th day (*p* < 0.05), 2–4 days later than all the other samples. This outcome is in agreement with a recent study reporting that the application of *L. paracasei* K5 in sourdough bread making showed a positive effect against rope spoilage delay [7]. This result can be also attributed to the high organic load of LP2 as it has been demonstrated previously [7,41,42,43].

### 3.5. Consumer Study

The results obtained through the preliminary sensory evaluation of the produced sourdough bread samples are presented in Table 4. All the sourdough breads produced in the present study were accepted by the consumers. In general, consumers commented that bread samples produced by the novel immobilized biocatalysts were characterized by acceptable bread appearance, texture, and smell. No statistically significant differences were observed for all the periods studied (1–5 days), except of the control sample that was scored lower by the consumers during the 2nd day of storage. Previous studies have examined the effect of wheat bran in bread making, concluding that it can affect the bread crumb textural properties but not the bread volume [44]. Likewise, in the present study no differences were observed regarding the volume and appearance of bread samples, while on the other hand the addition of wheat bran immobilized biocatalyst did not seem to affect the texture of sourdough breads compared to the control sample. 

## 4. Conclusions

The outcome of the present study showed that freeze-dried *L. paracasei* K5 and *L. bulgaricus* ATCC 11842 immobilized in WB retained their fermentative activity during sourdough bread production. Furthermore, the produced sourdough breads exhibited accepted sensorial properties and adequate profile regarding the volatiles content. Especially, sourdough bread made with immobilized freeze-dried *L. paracasei* K5 was more resistant against mold and rope spoilage, due to its higher organic load. Finally, it is of great importance that the cost of the proposed process is quite low since both LAB species were produced using cheese whey, which is a low-cost by-product of the dairy industry. 

## Figures and Tables

**Table 1:** Bacterial and yeast cell counts in sourdoughs.

Sourdough Sample	LAB	Yeast
	Log CFU/g	
A	7.3 ± 0.1	6.3 ± 0.2
B	9.0 ± 0.2	7.7 ± 0.2
C	7.3 ± 0.1	6.5 ± 0.2
D	8.9 ± 0.1	7.4 ± 0.1
E	7.3 ± 0.1	6.8 ± 0.1
F	8.9 ± 0.1	7.1 ± 0.1
C	8.9 ± 0.1	7.5 ± 0.1

**Table 2:** Physicochemical characteristics (acidity, volume, moisture loss, and organic acids content) and shelf-life of breads made with sourdoughs prepared with freeze dried *L. paracasei* K5 and freeze-dried *L. bulgaricus* ATCC 11842 as well as with the control sourdough.

Bread Sample	Sourdough Used	pH	TTA	SLV	Lactic acid	Acetic Acid	Formic Acid	Propionic Acid	*n*-Valeric Acid	Caproic Acid
			(mL 0.1 N NaOH)	(mL/g)	(g/g bread)	(g/kg bread)	(g/kg bread)	(g/kg bread)	(g/kg bread)	(g/kg bread)
LP1	A	4.70 ± 0.07 ^b^	7.13 ± 0.06 ^d^	2.35 ± 0.05 ^d^	2.25 ± 0.10 ^c^	0.92 ± 0.03 ^b^	0.05 ± 0.01 ^b^	0.03 ± 0.01 ^b^	0.04 ± 0.01 ^b^	0.04 ± 0.01 ^b^
LP2	B	4.51 ± 0.03 ^b^	8.67 ± 0.11 ^a^	2.62 ± 0.03 ^a^	2.90 ± 0.07 ^a^	1.11 ± 0.03 ^a^	0.09 ± 0.01 ^a^	0.06 ± 0.01 ^a^	0.07 ± 0.01 ^a^	0.07 ± 0.01 ^a^
LB1	C	4.55 ± 0.06 ^b^	7.50 ± 0.10 ^b^	2.25 ± 0.04 ^d^	2.48 ± 0.09 ^b^	0.35 ± 0.04 ^d^	0.04 ± 0.01 ^b^	Tr	Tr	Tr
LB2	D	4.51 ± 0.04 ^b^	8.10 ± 0.10 ^b^	2.19 ± 0.10 ^d^	2.72 ± 0.01 ^b^	0.41 ± 0.03 ^d^	0.06 ± 0.01 ^b^	0.03 ± 0.01 ^b^	Tr	0.03 ± 0.01 ^b^
LPB1	E	4.65 ± 0.04 ^b^	7.45 ± 0.06 ^c^	2.53 ± 0.05 ^c^	2.53 ± 0.07 ^b^	0.77 ± 0.03 ^c^	0.04 ± 0.01 ^b^	Tr	Tr	Tr
LPB2	F	4.56 ± 0.04 ^b^	7.10 ± 0.10 ^d^	2.49 ± 0.05 ^c^	2.63 ± 0.08 ^b^	0.82 ± 0.03 ^c^	0.06 ± 0.01 ^b^	0.03 ± 0.01 ^b^	0.03 ± 0.01 ^b^	0.03 ± 0.01 ^b^
W	G	4.89 ± 0.05 ^a^	6.50 ± 0.10 ^e^	2.55 ± 0.06 ^b^	1.86 ± 0.08 ^d^	0.94 ± 0.05 ^b^	0.06 ± 0.01 ^b^	0.03 ± 0.01 ^b^	0.03 ± 0.01 ^b^	0.03 ± 0.01 ^b^

TTA: Total Titratable Acidity; SLV: Specific Loaf Volume; Tr: Traces (<0.01 g/kg). Different superscript letters in a column indicate statistically significant differences (ANOVA, Duncan’s multiple range test, *p* < 0.05).

**Table 3:** Solid-phase microextraction (SPME) gas chromatography/mass spectrometry (GC/MS) analysis of aroma-related compounds (μg/g) extracted from breads made with sourdoughs prepared with freeze-dried *L. paracasei* K5 and freeze-dried *L. bulgaricus* ATCC 11842 as well as with the control sourdough.

KI	Compound	RI	Concentration (μg/g)			
			LP2	LB2	LPB2	W
***Alcohols***						
832	Ethanol	A	4.21 ± 0.18 ^a^	4.15 ± 0.15 ^a^	4.11 ± 0.17 ^a^	4.08 ± 0.11 ^a^
1012	Isobutyl alcohol	A	0.12 ± 0.02 ^b^	Tr	0.19 ± 0.02 ^a^	0.08 ± 0.01 ^c^
1120	Isoamyl alcohol	A	0.34 ± 0.09 ^b^	nd	0.34 ± 0.07 ^a^	0.17 ± 0.03 ^c^
1160	Butan-1-ol	A	0.20 ± 0.02 ^b^	0.05 ± 0.01 ^d^	0.19 ± 0.04 ^a^	0.13 ± 0.02 ^c^
1230	Pentan-1-ol	B	0.18 ± 0.02 ^a^	0.05 ± 0.01 ^c^	0.19 ± 0.04 ^a^	0.12 ± 0.01 ^b^
1257	Hexan-1-ol	A	0.11 ± 0.02 ^b^	0.10 ± 0.03 ^b^	0.15 ± 0.05 ^a^	0.11 ± 0.02 ^b^
1435	1-Heptanol	B	0.05 ± 0.01 ^b^	nd	0.09 ± 0.02 ^a^	nd
1466	Octan-1-ol	A	0.14 ± 0.02 ^a^	0.18 ± 0.04 ^a^	0.15 ± 0.05 ^a^	nd
1480	Heptan-2-ol	A	0.04 ± 0.01 ^b^	0.09 ± 0.01 ^a^	0.09 ± 0.02 ^a^	nd
1540	1-Octen-3-ol	B	0.18 ± 0.03 ^a^	0.05 ± 0.01 ^b^	0.16 ± 0.05 ^a^	nd
1670	Benzylalcohol	A	0.21 ± 0.05 ^a^	0.08 ± 0.01 ^b^	0.19 ± 0.04 ^a^	0.10 ± 0.02 ^b^
1812	2-Phenylethanol	A	0.37 ± 0.04 ^a^	0.22 ± 0.02 ^b^	0.33 ± 0.05 ^a^	0.19 ± 0.03 ^b^
***Esters***						
<800	Ethyl acetate	A	0.28 ± 0.05 ^a^	0.12 ± 0.04 ^b^	0.15 ± 0.02 ^b^	0.11 ± 0.03 ^b^
1107	Butyl acetate	A	0.13 ± 0.02 ^a^	0.07 ± 0.02 ^b^	Tr	0.06 ± 0.01 ^b^
1162	Hexyl acetate	B	0.09 ± 0.01 ^a^	nd	Tr	0.04 ± 0.01 ^b^
1250	Ethyl pentanoate	B	0.06 ± 0.01 ^a^	nd	0.03 ± 0.01 ^b^	0.03 ± 0.01 ^b^
1395	Ethyl exanoate	B	0.09 ± 0.01 ^a^	nd	0.07 ± 0.01 ^a^	nd
1438	Ethyl octanoate	B	0.08 ± 0.02 ^a^	nd	0.05 ± 0.01 ^a^	nd
1445	Ethyl heptanoate	A	0.07 ± 0.01 ^a^	nd	0.06 ± 0.01 ^a^	nd
1590	Isobutyl acetate	B	0.11 ± 0.01 ^a^	0.05 ± 0.01 ^b^	0.05 ± 0.01 ^b^	nd
1848	Ethyl dodecanoate	B	0.08 ± 0.01 ^a^	0.06 ± 0.01 ^a^	nd	nd
1850	2-Phenylethyl acetate	B	0.06 ± 0.01 ^a^	Tr	nd	nd
1925	Ethyl pentadecanoate	B	Tr	0.03 ± 0.01	nd	nd
2410	Ethyl octadecanoate	B	0.05 ± 0.01 ^b^	nd	0.08 ± 0.01 ^a^	Tr
2429	Ethyl 9-octadecenoate	B	0.05 ± 0.01 ^a^	nd	0.06 ± 0.01 ^a^	nd
***Carbonyl Compounds***						
<800	Acetaldehyde	B	0.33 ± 0.03 ^a^	0.23 ± 0.04 ^b^	0.17 ± 0.03 ^b^	0.08 ± 0.01 ^c^
812	Butanal, 2-methyl	B	0.05 ± 0.01 ^b^	0.09 ± 0.01 ^a^	0.11 ± 0.01 ^a^	0.04 ± 0.01 ^b^
986	Butanal, 3-methyl	A	0.06 ± 0.02 ^c^	0.25 ± 0.03 ^a^	0.13 ± 0.02 ^b^	0.06 ± 0.01 ^c^
1002	Hexanal	A	0.08 ± 0.01 ^b^	0.14 ± 0.02 ^a^	0.04 ± 0.01 ^c^	0.05 ± 0.01 ^c^
1080	Heptanal	A	Tr	0.11 ± 0.02 ^a^	0.08 ± 0.02 ^a^	Tr
1100	2,3-Butanedione	B	nd	0.07 ± 0.01	nd	nd
1334	Furfural	A	0.21 ± 0.02 ^a^	0.19 ± 0.03 ^a^	0.18 ± 0.02 ^a^	0.20 ± 0.04 ^a^
1358	Nonanal	B	0.27 ± 0.05 ^a^	0.05 ± 0.01 ^c^	0.10 ± 0.02 ^b^	Tr
1448	Butyrolactone	B	1.89 ± 0.15 ^a^	1.25 ± 0.05 ^b^	0.93 ± 0.02 ^c^	0.98 ± 0.12 ^c^
1458	Benzaldehyde	A	0.41 ± 0.07 ^a^	0.29 ± 0.02 ^b^	0.15 ± 0.02 ^c^	0.25 ± 0.03 ^b^
1541	2-Nonenal	B	0.13 ± 0.05 ^a^	nd	nd	0.05 ± 0.01 ^b^
1582	5-Methyl-furfural	B	0.28 ± 0.02 ^a^	0.05 ± 0.01 ^b^	nd	0.04 ± 0.01 ^b^

LP2: bread made with sourdough B, LB2: bread made with sourdough D, LPB2: bread made with sourdough F, W: bread made with control wild sourdough, KI: kovats index, RI: reliability of identification. A: Positive identification by MS data and retention times and those of standard compounds. B: Positive identification by MS data only. Tr: Compound present at <0.01 μg/g bread (traces); nd: not detected. Different superscript letters in a row indicate statistically significant differences (ANOVA, Duncan’s multiple range test, *p* < 0.05).

**Table 4:** Preliminary sensory evaluation test of breads made with sourdoughs prepared with freeze dried *L. paracasei* K5 and freeze-dried *L. bulgaricus* ATCC 11842 as well as with the control sourdough.

Bread Sample	Storage Time (Days)					
	0	1	2	3	4	5
LP1	8.8 ± 0.2 ^a^	8.3 ± 0.2 ^a^	7.7 ± 0.2 ^a^	7.0 ± 0.2 ^a^	6.2 ± 0.1 ^a^	5.3 ± 0.2 ^a^
LP2	8.9 ± 0.1 ^a^	8.2 ± 0.1 ^a^	8.0 ± 0.1 ^a^	7.2 ± 0.2 ^a^	6.4 ± 0.1 ^a^	5.4 ± 0.1 ^a^
LB1	8.7 ± 0.2 ^a^	8.4 ± 0.2 ^a^	7.8 ± 0.2 ^a^	7.0 ± 0.1 ^a^	6.2 ± 0.1 ^a^	5.3 ± 0.1 ^a^
LB2	8.7 ± 0.1 ^a^	8.4 ± 0.1 ^a^	7.9 ± 0.1 ^a^	7.1 ± 0.1 ^a^	6.2 ± 0.2 ^a^	5.4 ± 0.1 ^a^
LPB1	8.7 ± 0.2 ^a^	8.1 ± 0.2 ^a^	8.0 ± 0.2 ^a^	7.0 ± 0.1 ^a^	6.2 ± 0.1 ^a^	5.5 ± 0.1 ^a^
LB2	8.6 ± 0.1 ^a^	8.3 ± 0.1 ^a^	8.0 ± 0.1 ^a^	7.2 ± 0.1 ^a^	6.2 ± 0.1 ^a^	5.4 ± 0.2 ^a^
W	8.7 ± 0.2 ^a^	8.1 ± 0.1 ^a^	7.0 ± 0.2 ^b^	6.0 ± 0.2 ^b^	5.0 ± 0.1 ^b^	4.8 ± 0.2 ^b^

LP1: bread made with sourdough A, LP2: bread made with sourdough B, LB1: bread made with sourdough C, LB2: bread made with sourdough D, LPB1: bread made with sourdough E, LPB2: bread made with sourdough F, W: bread made with control wild sourdough. Different superscript letters in a row indicate statistically significant differences (ANOVA, Duncan’s multiple range test, *p* < 0.05).
